# Assessment of Oxidative Stress and DNA Damage in Dogs with Visceral Leishmaniasis Using Urinary 8-Hydroxy-2′-Deoxyguanosine

**DOI:** 10.3390/vetsci13030230

**Published:** 2026-02-28

**Authors:** Demet Derya, Songul Erdogan, Tahir Ozalp, Hasan Erdogan, Serdar Pasa, Mehmet Gultekin, Kerem Ural, Ilia Tsachev

**Affiliations:** 1 Department of Internal Medicine, Veterinary Faculty, Aydın Adnan Menderes University, Aydın 09016, Türkiye; 2320600107@stu.adu.edu.tr (D.D.); tozalp@adu.edu.tr (T.O.); hasan.erdogan@adu.edu.tr (H.E.); spasa@adu.edu.tr (S.P.); mgultekin@adu.edu.tr (M.G.); kural@adu.edu.tr (K.U.); 2 Department of Clinical Science, Faculty of Veterinary Medicine, Medical University-Pleven, 5800 Pleven, Bulgaria; iliya.tsachev@trakia-uni.bg; 3 Faculty of Veterinary Medicine, Trakia University, 6000 Stara Zagora, Bulgaria; 4 Bulgarian Academy of Sciences, 1000 Sofia, Bulgaria

**Keywords:** biomarker, dog, DNA damage, oxidative stress, vector born disease

## Abstract

Canine leishmaniasis (CanL) is a major vector-borne disease with zoonotic potential, and oxidative stress plays an important role in its pathogenesis. CanL is a chronic, progressive disease in which clinical signs may develop gradually over months or even years, depending on host immune response and parasite burden. This study aimed to evaluate oxidative DNA damage in dogs with CanL by measuring urinary 8-hydroxy-2′-deoxyguanosine (8-OHdG) levels and oxidative stress markers. A total of 54 dogs, including 34 dogs with CanL and 20 healthy controls, were included. Urinary 8-OHdG and serum malondialdehyde (MDA) were measured using ELISA method, whereas serum TAC and TOC were determined using automated colorimetric method. Dogs with CanL showed significantly higher TAC (1.30 ± 0.05 vs. 1.04 ± 0.06 mmol/L, *p* = 0.001) and TOC levels (18.91 ± 3.29 vs. 8.23 ± 1.61 μmol H_2_O_2_ Eq/L, *p* = 0.002) compared with healthy controls. Although urinary 8-OHdG and MDA levels were higher in infected dogs, these differences were not statistically significant (*p* > 0.05). These findings indicate a disturbance in oxidative balance in dogs with CanL, characterized by simultaneous increases in both oxidant and antioxidant parameters, while urinary 8-OHdG, as measured by ELISA, may have limited ability to reflect cellular oxidative DNA damage in dogs with CanL, rather than indicating an absence of DNA damage.

## Simple Summary

Canine leishmaniasis is an important parasitic disease that affects dogs and can also be transmitted to humans. This disease can cause oxidative stress, which may damage cells and DNA. In this study, we evaluated oxidative stress and DNA damage in dogs with leishmaniasis by measuring oxidative stress markers in blood and a DNA damage marker in urine. We found that dogs with leishmaniasis exhibited alterations in oxidative balance, characterized by increased total oxidant capacity (TOC) along with a concurrent increase in total antioxidant capacity (TAC), but urinary DNA damage levels were not significantly different from healthy dogs. These results suggest that oxidative stress is involved in the disease, while urinary DNA damage markers measured by ELISA may not be reliable diagnostic tools.

## 1. Introduction

*Leishmania infantum*, an obligate intracellular parasite, is a prevalent etiological agent of canine leishmaniasis (CanL) in dogs. CanL is a significant vector-borne disease with zoonotic potential, transmitted by sand flies [1]. Disease is universally endemic in some European parts (Türkiye/Bulgaria), particularly in tropical and subtropical nations, including Türkiye [2,3,4,5]. Clinical manifestations can range from self-limiting cutaneous findings to life-threatening organ failure, depending on the parasite and the host immune response [6]. Among these, the visceral form represents the most severe form in mammalians [7]. The progression and severity of the disease largely depend on the host immune response. Oxidative stress is closely associated with this process and is considered primarily a consequence of pathogen-induced inflammatory and immune responses rather than the initiating cause of the disease.

The excessive production of oxidants, exceeding the neutralizing capacity of antioxidants, results in oxidative stress and consequently cellular damage. Distribution of this balance is a main factor in the pathogenesis of diseases [8]. The oxidative stress is associated with progression of CanL [9,10,11,12]. There is complex communication between host immune response, life of the parasite, and oxidative stress. *Leishmania* spp. can initially escape the immune response by inhibiting reactive oxygen species (ROS) formation in phagocytes; nevertheless, the ensuing inflammation in CanL is marked by an enhanced influx of activated immune cells that produce elevated amounts of oxidants. This facilitates disease progression and a simultaneous reduction in antioxidant defenses [11,12,13]. Following these pathological process, oxidative markers increase in the blood and tissues and other biological fluids are also affected [9,14,15]. Numerous studies have shown that the balance between the oxidative status and antioxidative status of disease process is revealed by elevated levels of circulating oxidation markers [14,16,17,18] and fluctuating alterations in antioxidant markers [14,19,20].

Evaluation of total antioxidant capacity (TAC) and total oxidant (TOC) levels is a common diagnostic procedure for determining oxidative status [21]. Additionally, malondialdehyde (MDA) is used as a marker of lipid peroxidation [22]. 8-OHdG is a sign of oxidative DNA damage. It is an oxidative product that forms when hydroxyl radicals react with the guanine base at the eighth position [23]. 8-OHdG is the most well-known biomarker of nucleic acid oxidation. It is released in urine when DNA damage is fixed [24,25]. The measurement of 8-OHdG in urine is thought to be a good way to monitor systemic oxidative stress in living things because it is not invasive and is not very complicated [25,26]. Moreover, 8-hydroxy-2′-deoxyguanosine (8-OHdG) indicates oxidative DNA damage to be identified in tissues and biological fluids [27,28,29,30]. It is most commonly measured in urine, as urinary 8-OHdG is considered more stable than plasma levels, exhibits lower day-to-day variability, and reflects systemic oxidative stress in a non-invasive manner. CanL is a chronic and progressive disease in which renal involvement is frequent, particularly in advanced stages, often leading to immune-complex glomerulonephritis and chronic kidney damage. Therefore, assessment of urinary oxidative DNA damage markers may provide additional insight into systemic and renal oxidative processes in CanL [7].

The present study aimed to evaluate oxidative DNA damage in dogs with CanL by measuring urinary 8-OHdG levels. In addition, serum MDA, TAC, and TOC were determined to assess systemic oxidative status and to better characterize oxidative imbalance in CanL.

## 2. Materials and Methods

### 2.1. Animals

The study was conducted on 54 dogs including 34 infected with *Leishmania* spp. and 20 healthy dogs, presented to the Department of Internal Medicine, Faculty of Veterinary Medicine, Adnan Menderes University, Aydın.

Dogs with CanL were subject to the following inclusion criteria:

- Being older than one year of age;
- Have clinical findings of multiple dermatological lesions including pruritus, alopecia, generalized lymphadenopathy, ulcerative exfoliative dermatitis around the nose and eyes, and onychogryphosis;
- Being mono-infected (serologically determined by rapid test kit and IFAT) and negative for other vector-borne diseases;
- Not being pregnant or lactating;
- Not having received leishmaniasis treatment within the last two years but having been diagnosed previously.

The control group comprised healthy dogs which were also selected based on the criteria of being ≥one year old, having no pathological findings, not being pregnant or lactating, and having no known ongoing chronic disease.

**Figure 1:**
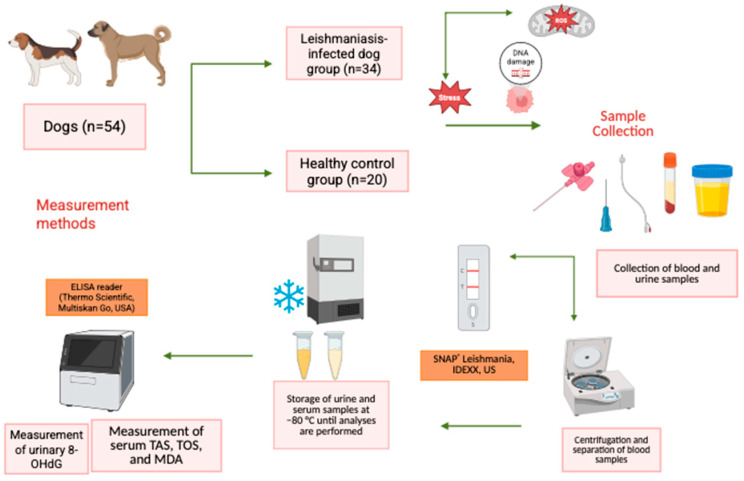
Workflow diagram.

The workflow diagram in Figure 1 was followed for study.

### 2.2. Diagnosis

The evaluation of leishmaniasis was based on the presence of clinical signs, hematological and biochemical parameters, a positive result from a rapid diagnostic test kit (SNAP^®^ Leishmania, IDEXX, Westbrook, ME, USA), and an anti-leishmanial antibody titer of ≥1/64 determined by IFAT. Complete blood count (CBC) parameters including red blood cell count (RBC), white blood cell count (WBC), hemoglobin concentration (Hb), hematocrit (HCT), and platelet count (PLT) were assessed. Serum biochemical analysis included urea, creatinine, total protein, albumin, and globulin levels.

### 2.3. Sampling

Blood samples were obtained via the *V. cephalica* or *V. saphena* and placed into both heparinized and serum tubes. For routine evaluation, complete blood count and biochemical analyses were performed using the anticoagulated blood samples. Serum tubes were centrifuged at 1800× *g* for 10 min to separate the serum; 0.5 mL aliquots were transferred into eppendorf tubes and frozen for IFAT analysis. The remaining serum samples were stored at −20 °C until analysis of oxidative stress markers.

For the determination of urinary 8-OHdG levels, urine samples were collected in sterile containers. After discarding the initial portion by catheterization, at least 5 mL of urine was obtained. Urine samples were collected by sterile urethral catheterization using standard clinical procedures. The first portion of urine was discarded to minimize potential contamination and epithelial cell interference. All samples were visually examined, and samples with macroscopic hematuria were excluded. The same sampling protocol was applied to both infected and control dogs. The collected urine samples were stored at −80 °C until analysis.

### 2.4. Determination of Oxidative Stress Markers

MDA, TAC, and TOC were determined using commercially available ELISA kits (Rel Assay Diagnostics, Gaziantep, Turkey). Measurement of MDA levels were as follows: 50 μL of standards and blood sample was added to the wells, followed by 50 μL of biotinylated antigen solution and 50 μL of Horseradish Peroxidase HRP. The plates were incubated at 37 °C for 60 min and washed five times before adding 50 μL of Substrate A (tetramethylbenzidine) and B (hydrogen peroxide). Consequently, the sample was incubated in the dark for 10 min and the reaction was stopped with 50 μL of stop solution. Lastly, the absorbance was read at 450 nm using a spectrophotometer. Serum TAC was measured with description of the study presented by Erel [31]. In this method, hydroxyl radicals generated by the Fenton reaction react with o-dianisidine to form a colored diazinyl radical, which is suppressed by serum antioxidants. The results were expressed as mmol Trolox Eq/L.

TOC was determined with a commercial kit (Rel Assay Diagnostics, Gaziantep, Turkey) based on the method of Erel [32].

The assay is based on the oxidation of ferrous to ferric ions in an acidic medium and the subsequent formation of a colored complex with a chromogen. The absorbance was measured at 660 nm, and TOC values were calculated as μmol H_2_O_2_ Eq/L. Serum TAC, TOC, and MDA levels were reported in mmol/L, µmol/L, and nmol/L, respectively.

### 2.5. Urinary 8-Hydroxy-2′-Deoxyguanosine

Urinary 8-OHdG concentrations were measured using a commercial ELISA kit (BT LAB Canine 8-Hydroxy-desoxyguanosine, Cat. No. EA0062Ca, BT LAB, Shenyang, China), and the analyses were performed in a specialized laboratory under following standardized methods. The samples were added to pre-coated microtiter plates, followed by the addition of a biotinylated antigen. Antigens present in the samples competed with the biotinylated antigen for binding to the capture antibody, and the plates were incubated. Unbound antigen was removed during the washing step.

Subsequently, avidin- (HRP) was added, followed by another incubation. Excess avidin-HRP was eliminated by washing, after which TMB substrate was introduced, leading to the development of color. The reaction was stopped by adding an acidic stop solution, which changed the color to yellow.

The optical density was then measured at 450 nm. The intensity of the developed color was inversely proportional to the concentration of 8-OHdG in the samples. Final concentrations were determined by comparing the optical density values of the samples with a standard calibration curve. Urinary 8-OHdG concentrations were expressed as pg/mL.

### 2.6. Statistical Analysis

Statistical analyses were performed using the SPSS software programme, version 16 (SPSS Inc., Chicago, IL, USA). The normality of data distribution was assessed using the Shapiro–Wilk test. Differences between groups not following a normal distribution were analyzed using the Kruskal–Wallis variance analysis. For normally distributed data, one-way ANOVA was applied, and post hoc comparisons were conducted using Duncan’s test. To determine the specific group or groups responsible for significant differences, the Bonferroni-corrected Mann–Whitney U test was employed. *p* < 0.05 was considered statistically significant. All data were expressed as mean ± standard error (SE).

## 3. Results

### 3.1. Clinical Findings

**Figure 2:**
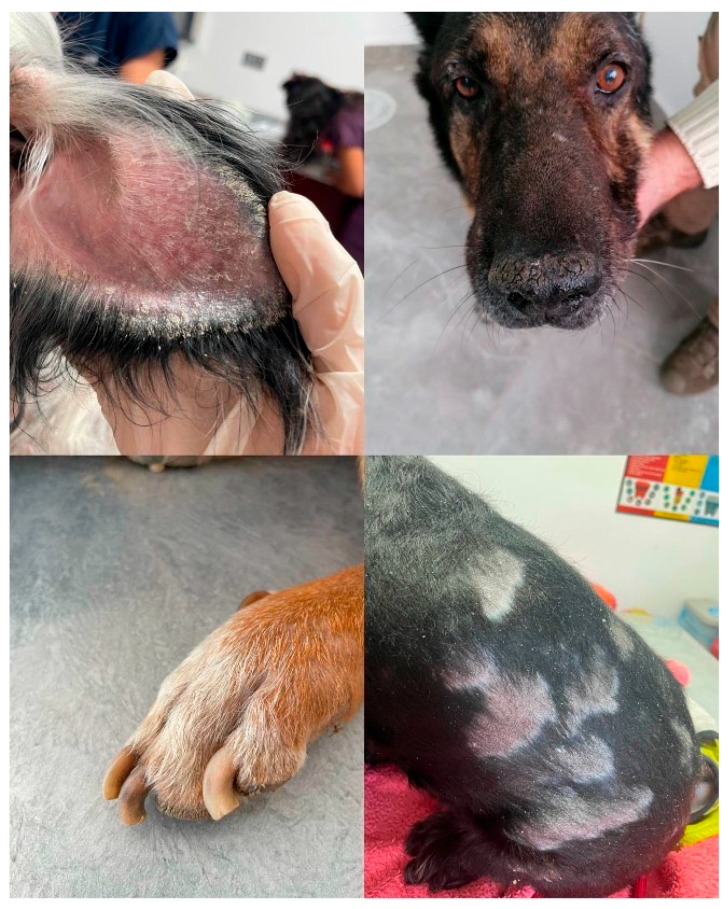
Common dermatological findings observed in infected dogs.

Among the infected dogs, dermatological lesions were the most observed clinical findings. The most frequent symptoms included systemic signs lymphadenopathy (64.7%), and dermatological signs desquamation (55.88%), nasal hyperkeratosis (38.23%), onychogryphosis (35.29%), and ear tip necrosis (29.41%). Less commonly observed findings included systemic and ocular signs weight loss (14.70%), loss of appetite (17.64%), lethargy (14.70%), epistaxis (14.70%), as well as dermatological findings periocular alo-pecia, hyperkeratosis (26.47%), and alopecia (17.64%) (Figure 2).

### 3.2. Urinary 8-OHdG and Blood TAC, TOC, MDA

The mean urinary 8-OHdG and serum MDA levels were 1043.79 ± 78.96 pg/mL, 10.50 ± 2.08 nmol/L in the infected group and 918.79 ± 105.76 pg/mL, 8.38 ± 2.22 nmol/L in the control group, respectively (Table 1). Although a higher mean value of both parameters was observed in the infected group, the difference was not statistically significant (*p* = 0.333 and *p* = 0.352). Additionally, TAC and TOC levels were found to be significantly higher in the infected dogs compared with the control group (*p* = 0.001).

## 4. Discussion

The present study aimed to evaluate oxidative stress and DNA damage in dogs with CanL by measuring urinary 8-OHdG and blood oxidative stress markers, including TAC, TOC, and MDA. Our findings revealed that TAC and TOC levels were significantly increased in infected dogs compared with controls; however, urinary 8-OHdG and MDA levels were found to be insignificantly increased. Although TAC and TOC are generally considered opposing parameters, their simultaneous elevation does not represent a contradiction. Increased TOC reflects enhanced oxidant production, whereas elevated TAC may indicate a compensatory upregulation of antioxidant defenses in response to an oxidative challenge. Therefore, the concurrent increase in both parameters likely reflects an activated but imbalanced redox regulatory system in dogs with CanL.

CanL is a systemic condition that may affect any organ, tissue, or biological fluid, presenting with nonspecific clinical symptoms. Diverse clinical analyses have been documented in various native processors. Clinically, it manifests as anemia, emaciation, splenomegaly, local or generalized lymphadenopathy, cutaneous-ocular lesions, weight loss, and cachexia [33,34]. Monteiro et al. [35] documented that 41 of 185 adult dogs exhibited alopecia, xerosis, and dermatological abnormalities including nodules and ulcerations, in addition to emaciation and ocular disorders. Meléndez-Lazo et al. [34] documented skin lesions in 78.4%, lymphadenopathy in 64.7%, and weight loss in 47.1%. Silva et al. [36] found that of 265 dogs afflicted with Canine Visceral Leishmaniasis (CVL), the predominant infections observed were lymphadenomegaly, nasal and ear lesions, nutritional deficiencies, alterations in skin manifestation, pallor, onychogryposis, nasal depigmentation, and ocular complications; it was particularly noted that onychogryposis, nasal depigmentation, and keratoconjunctivitis exhibit significant correlations with CVL. Our study noted that the dogs predominantly exhibited clinical symptoms of dermatological origin. Lymphadenopathy (64.7%) was notably prevalent among systemic lesions, whereas desquamation (55.88%), nasal hyperkeratosis (38.23%), onychogryposis (35.29%), and ear tip necrosis (29.41%) were significant among dermatological lesions. Systemic, ocular, and cutaneous manifestations were observed in smaller percentages, including weight loss (14.70%), loss of appetite (17.64%), weakness (14.70%), epistaxis (14.70%), hair loss in periocular regions, hyperkeratosis (26.47%), and local alopecia (17.64%).

Oxidative stress is characterized by an imbalance between the production of ROS and the antioxidant mechanisms that safeguard the organism against these chemical assaults. The unregulated excess of the ROS can result in protein and lipid peroxidation, as well as DNA damage, ultimately leading to cellular and tissue death [37]. In recent years, oxidative stress has been examined across numerous disease situations in multiple animal species and is pivotal in the manifestation of clinical symptoms [11,38]. In leishmaniasis, following the phagocytosis of parasites by macrophages, these cells generate different ROS as a host defensive mechanism [39]. This initiates signaling pathways linked to inflammation and immunological responses [40,41]. Elevated oxidative damage in parasite illnesses is believed to stem from macromolecules resulting from lipid peroxidation and oxidative DNA damage, which represent distinct clinical states [42]. Kiral et al. [15] conducted a study that indicated elevated oxidative damage indicators and diminished antioxidant responses in dogs afflicted with leishmaniasis. Following phagocytosis of leishmania parasites, macrophages generate ROS as part of the host defense response [39]. The significantly elevated serum TAS and TOS levels observed in infected dogs (*p* < 0.005) indicate activation of the redox regulatory system during disease pathogenesis. Increased TOS levels reflect enhanced oxidant burden associated with parasite-induced inflammatory activation, whereas elevated TAS levels may represent a compensatory upregulation of antioxidant defense mechanisms in response to oxidative stress. In fact, the study by Gültekin et al. [43] also found that TOS levels stayed high throughout the disease. In this research conducted by Gültekin et al. [43], TOC values were significantly elevated (*p* < 0.001) in dogs with Visceral Leishmaniasis relative to healthy dogs, whereas no notable change was detected in TAC values between the two groups. In the study by Kıral et al. [15] involving 10 clinically healthy dogs and 15 dogs with leishmaniasis aged 2 to 6 years, MDA levels, a marker of lipid peroxidation, were considerably elevated in infected dogs relative to the control group (*p* < 0.05). Conversely, TAC levels were seen to be reduced in dogs with leishmaniasis (*p* < 0.05). Consistent with findings in veterinary literature, Serarslan et al. [39] observed markedly elevated serum MDA and nitric oxide levels in patients with cutaneous leishmaniasis relative to those who were treated.

Studies have reported in CanL, supporting its role as a marker of oxidative DNA damage. Previously oxidative DNA damage was presented in infected dogs as shown by increased 8-OHdG levels measured with comet assays [15] and both comet and micronucleus tests [44]. These findings indicate the potential role of 8-OHdG in the genotoxic processes of CanL. Contrarily, our study did not detect significant differences in urinary 8-OHdG between infected and control dogs. This diversity may be explained by the compartmentalization of DNA damage within blood cells or tissues, as shown by Ruiz et al. [44]. In that study, peripheral blood assays detected DNA damage, whereas urinary biomarkers did not. It may also reflect differences in disease stage or parasite burden [15] or variability in renal excretion of 8-OHdG in chronic kidney disease in cats, where plasma 8-OHdG increased significantly without the urinary 8-OHdG/creatinine ratio [45]. In our investigation, urinary 8-OHdG and serum MDA levels were numerically elevated in infected dogs compared with healthy controls; however, these differences did not reach statistical significance (*p* > 0.05). Although these findings are partially consistent with previous reports demonstrating increased oxidative DNA damage in CanL, the lack of statistical significance in our cohort suggests that oxidative stress may not have exceeded the threshold required to induce persistent macromolecular injury detectable in urine. Similar elevations in 8-OHdG and MDA have been reported in inflammatory and infectious conditions such as pyoderma [12], venereal tumor [13], and *Babesia vogeli* infection [11]. Moreover, Kiral et al. [15] demonstrated oxidative DNA damage in lymphocytes using comet assays, indicating that cellular genotoxicity may occur independently of urinary biomarker expression. Therefore, the absence of significant urinary differences does not exclude the presence of oxidative DNA injury but may instead reflect tissue-specific localization of damage or variability in renal excretion dynamics.

Conversely, TAC and TOC values, critical parameters of oxidative balance, exhibited a significant elevation (*p* < 0.005) in the infected group relative to the control group, indicating that the infection activates the antioxidant defense system, leading to an augmented systemic antioxidant capacity. Taken together, these findings suggest that urinary 8-OHdG may not fully reflect cellular oxidative DNA damage at the cellular level, as previously demonstrated using comet and micronucleus assays [15,44]. This discrepancy may be explained by the compartmentalization of DNA damage within cells and its variable renal excretion, which may prevent consistent reflection of cellular DNA damage in urinary biomarkers, as reported previously [44,45]. Therefore, longitudinal monitoring of oxidative DNA damage markers may provide clinically relevant prognostic information in dogs with CVL.

## 5. Conclusions

A limitation of this study is the small sample size, and that only urinary 8-OHdG was evaluated. The use of complementary assays, such as comet or micronucleus tests, could provide a more extensive evaluation of oxidatively induced DNA damage. Additionally, dogs were not stratified according to a standardized clinical severity scoring system. Future studies evaluating oxidative stress parameters across different clinical stages of CanL would help clarify potential associations between disease severity and oxidative imbalance. Also urinary 8-OHdG levels were not normalized to urinary creatinine. Future studies should consider expressing results as 8-OHdG/creatinine ratios to minimize variability related to urine concentration.

In conclusion, dogs with CanL exhibited significant alterations in serum oxidative stress parameters, characterized by increased TAC and TOC levels, reflecting systemic oxidative imbalance. However, urinary 8-OHdG measured by ELISA did not prove to be a reliable indicator of oxidative DNA damage in CanL. Larger-scale studies integrating multiple oxidative markers are warranted to explain their clinical relevance and potential diagnostic value.

## Figures and Tables

**Table 1:** Comparison of oxidative stress parameters and DNA damage indicators between groups.

Parameter	Infected Group	Control Group	*p* Value
	Mean ± SE		
8-OHdG (pg/mL)	1043.80 ± 78.96	918.79 ± 105.76	0.333
TAC (mmol/L)	1.30 ± 0.05	1.04 ± 0.06	0.001
TOC (μmol/L)	18.91 ± 3.29	8.23 ± 1.61	0.002
MDA (nmol/L)	10.51 ± 2.08	8.38 ± 2.22	0.352

## Data Availability

The data presented in this study are available on request from the corresponding author due to ethical restrictions and the need to protect animal owner confidentiality.

## Abbreviations

The following abbreviations are used in this manuscript:

8-OHdG	8-hydroxy-2′-deoxyguanosine
CanL	Canine leishmaniasis
MDA	Malondialdehyde
ROS	Reactive oxygen species
TAC	Total antioxidant capacity
TOC	Total oxidant capacity
